# Contraceptive Use and Method Preferences among HIV Positive Women in Ethiopia: A Systematic Review and Meta-analysis

**DOI:** 10.1155/2020/6465242

**Published:** 2020-09-18

**Authors:** Getnet Gedefaw, Adam Wondmieneh, Asmamaw Demis

**Affiliations:** ^1^Department of Midwifery, College of Health Sciences, Woldia University, P.O. Box: 400, Woldia, Ethiopia; ^2^Department of Nursing, College of Health Sciences, Woldia University, P.O. Box: 400, Woldia, Ethiopia

## Abstract

**Background:**

Preventing unintended pregnancies among HIV positive women has a vital role to prevent mother to child transmission. Besides, increasing access to contraceptives has a number of economical importance and reducing the costs for mitigating the unintended pregnancy consequences. Therefore, this study is aimed at assessing the contraceptive use and method of preference among HIV positive women in Ethiopia.

**Methods:**

A systematic review and meta-analysis reporting guideline was applied. Articles searched from the Scopus, Pubmed/MEDLINE, EMBASE, AJOL, Hinari, and Google scholar were included in this review. The Stata 11 software was used to compute the analysis. Heterogeneity of the studies was detected using the Cochran *Q* test and *I*^2^ test statistics. Egger's test was used to check the evidence of publication bias within the studies. Subgroup analysis and sensitivity analysis was computed with the evidence of heterogeneity.

**Results:**

Ten thousand one hundred twenty one (10121) women living with HIV/AIDS were recruited in this study. The national estimated prevalence of contraceptive use among HIV positive women in Ethiopia was 57.78% (95% CI: 48.53-67.03). Injectables and male condom were the most preferred contraceptives accounted for 36.00% (95% CI: 6.64-45.35) and 32.74% (95% CI: 21.08-44.40), respectively. Discussion with husband/partner (AOR: 4.70, 95% CI: 2.18-10.12), disclosure of HIV status to spouse/partner (AOR: 2.18, 95% CI: 1.55-3.06), ever counseled for modern contraceptives (AOR: 2.79, 95% CI: 2.01-3.88), attending secondary and above education (AOR: 3.12, 95% CI: 2.15-4.51), and having more than one live child (AOR: 2.61, 95% CI: 1.86-3.66) were increasing the likelihood of contraceptive use whereas not currently married women (AOR: 0.23, 95% CI: 0.16-0.34) was decreases the odds of contraceptive use.

**Conclusion:**

In Ethiopia, more than half of the women living with HIV/AIDS were using contraceptives. Discussion with husband/partner, disclosure of HIV status to spouse/partner, ever counseled for modern contraceptives, attending secondary and above education, and having more than one live child were increasing the uptake of contraceptives among HIV positive women. Partner discussion, having adequate information towards contraceptive use, and having desired number of child could increase the utilization; as a result, obstetric complication with HIV positive women due to unintended pregnancy is significantly decreasing.

## 1. Background

Human immunodeficiency virus (HIV) is the most common public health issue and destructive epidemics that the world has ever faced. According to UNAIDS 2019 report, globally 37.9 million people were living with HIV and 1.7 million people became newly infected with HIV as well as around 770000 people died from AIDS-related illnesses. Around 6000 young women among age group of 15-24 years become infected with HIV every week. However, in sub-Saharan Africa, four in five new infections among adolescents aged 15–19 years are in girls. Worldwide, the epidemiology of new HIV infections is 54% varied across the countries in the range of 25%-95% in Eastern Europe and Central Asia, Middle East and North Africa, Western and Central Europe and North America, Asia and the Pacific, and Eastern and Southern Africa [1].

Different literatures showed that unintended pregnancy has emergency obstetric complications for women living with HIV/AIDS; furthermore, the magnitude of unintended pregnancy is 96%, 71%, and 43% in Thailand, South Africa, and Uganda [2–4]. According to World Health Organization (WHO) comprehensive prong strategy, prevention of mother-to-child transmission of HIV/AIDS is the first strategy to prevent the vertical transmission of HIV to her baby; moreover, this strategy could be achieved through increasing access to and use of effective contraception [5, 6].

A study conducted in sub-Saharan African population showed that contraceptive strategy averts 28.6% more HIV positive births than Neverapine for PMTCT, furthermore increasing access to contraception and preventing unintended pregnancies, integration reduces new and opportunistic pediatric HIV infections, and the number of children needing HIV treatment, care, and support [7, 8].

According to the 2016 Ethiopia demographic and health survey report, one in every three currently married women used modern contraceptive. Besides, injectable contraceptive method, long acting or permanent methods, and condom constituted 23%, 8%, and 4%, respectively [9].

In Ethiopia, different studies have been showed variation in the magnitude of contraceptive use among women living with HIV accounted for 30.3% in Amhara region, 39% Addis Ababa, 46% Tigray region, 64.1% southern Ethiopia, and 50% in Gondar University specialized hospital [10–14], whereas injectable, male condom, implant, IUD, and oral contraceptives were the most commonly used and preferred methods of contraceptive [11–14].

Uptake of contraception helps women avoid unplanned or unwanted pregnancies and prevent unsafe abortions. A systematic review and meta-analysis study showed that HIV positive women have eight times the risk of a pregnancy-related death compared to women without HIV infection. Besides, contraceptive use helps the women to space and limit the births, which benefit the health of the mother and her baby [15].

Various factors such as sociocultural norms and traditions, lack of comprehensive knowledge on contraceptive methods, lack of advice from health professionals, not start ART, fear of side effects, illiteracy, and inaccessibility of preferred contraceptives limit women living with HIV to seek and use modern contraceptive service and information [16, 17].

Even though there are different single studies reported the prevalence of contraceptive use among HIV positive women and its associated factors, there are no national studies which show the nationwide magnitude of contraceptive use among women living with HIV/AIDS in Ethiopia. Therefore, this systematic review and meta-analysis is aimed at estimating the pooled prevalence of contraceptive use and method of preferences among women living with HIV/AIDS in Ethiopia.

## 2. Method and Materials

### 2.1. Search Strategy

Articles accessed in the Scopus, Pubmed/MEDLINE, Hinari, EMBASE, Google scholar, African journals, and online university repositories were considered in this systematic review and meta-analysis (Table 1). Different MeSH terms and search engines including “contraceptive use” OR “uptake of contraceptive” OR “utilization of family planning,” OR “modern contraceptive use” OR“ contraceptive preference,” OR “met need of family planning,” OR “uptake of family planning” AND “among women living with HIV/AIDS,” OR “among sexually active women living with HIV/AIDS” AND related in Ethiopia.

### 2.2. Eligibility Criteria

#### 2.2.1. Inclusion Criteria

*(1) Study Design*. Cross-sectional studies reported the prevalence or widely used contraceptives and risk factors for contraceptive use among HIV positive women were included.

*(2) Language*. Only English language literature and research articles were included.

*(3) Publication*. Both published and unpublished research articles were used.

*(4) Searching Date*. Articles searched from April 1-30, 2020 were included.

*(5) Study Population*. Women living with HIV/AIDS were included in this study.

#### 2.2.2. Exclusion Criteria

Articles without full text and abstract, duplicated studies, anonymous reports, and editorial reports were excluded.

### 2.3. Data Extraction and Quality Assessment

The Standard Microsoft Excel spread sheet was used to export data from online databases. Two authors (GG and AD) were independently extracted and reviewed all the articles included in this study. Any disagreement was handled by the third reviewer (AW). After all, a consensus was reached through discussion between authors. The Newcastle-Ottawa Quality Assessment Scale (NOS) for cross-sectional studies was used to assess the methodological quality of each study (sampling strategy, sample size, and representativeness of the study), comparability, and measurement of outcomes [18]. Each study scored ≥ 7 out of 10 of the NOS criteria was considered as having good quality (Table S1). This cutoff point was declared after reviewing relevant piece of literatures. All authors independently assessed the articles for the consideration and inclusion for the study.

#### 2.3.1. Measurement of Outcomes

This systematic review and meta-analysis had two main outcome measurements. Contraceptive use was the first outcome of the study whereas method of preference and predictors of contraceptive use among HIV positive women were the second outcome of this review. The pooled prevalence and adjusted odds ratio were calculated for risk factors reported in the study.

*(1) Contraceptive Use*. Contraceptive use is the current use of any modern method by women to delay or avoid unintended pregnancy for the last one month.

*(2) Modern Methods*. The modern methods are oral pills, intrauterine device, injectable, implants, male condom, female condom, and female and male sterilization.

#### 2.3.2. Data Processing and Analysis

The Microsoft Excel (2016) and STATA version 11 software were used for the data entry and analysis, respectively. The funnel plot and Egger's regression test were conducted to check potential publication bias [19, 20]. The Cochrane *Q* test and *I*^2^ with its corresponding *p* value were used to assess the heterogeneity of the study [21, 22]. Hence, there was marked heterogeneity within studies; the random effect model was used to compute the pooled prevalence of intestinal parasitic infection among HIV/AIDS patients. Furthermore, for the evidence of heterogeneity within studies subgroup and sensitivity analysis was computed. Moreover, the estimated pooled prevalence rate was reported with a 95% confidence interval (CI), and *p* value < 0.05 was considered statistically significant. The odds ratio was calculated to measure the association between the outcome variable and the determinants.

## 3. Results

### 3.1. Characteristics of the Included Studies

We retrieved 577 studies from the PubMed/MEDLINE, HINARI, EMBASE, Scopus, Google Scholar and African Journals, and online university repository research articles. After duplicates were expunged, 234 studies remained.

**Figure 1:**
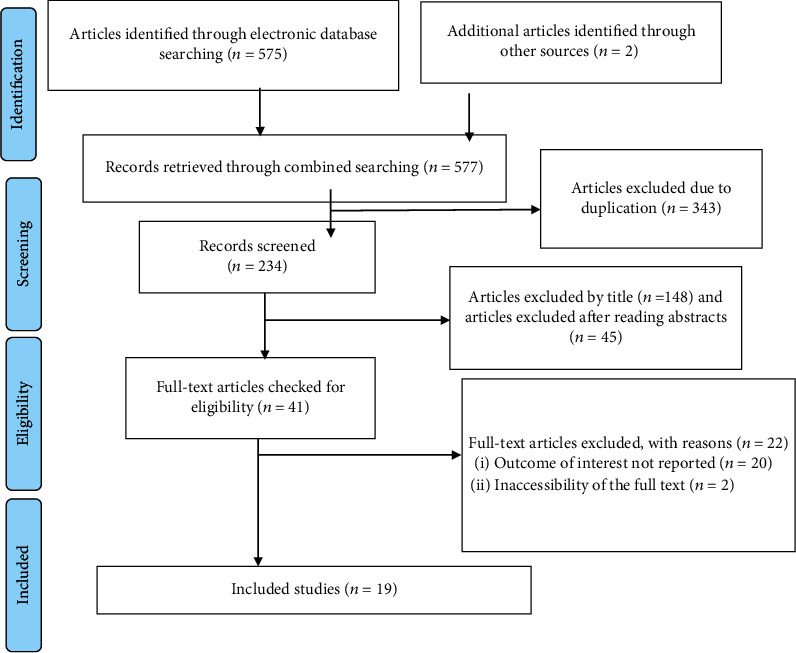
Flow chart of selection for systematic review and meta-analysis of contraceptive use among HIV positive women in Ethiopia.

Out of the remaining 234 articles, 193 articles were excluded after review of their titles and abstracts. Therefore, 41 full-text articles were accessed and assessed for inclusion criteria, which resulted in the further exclusion of 22 articles. Out of these, 20 studies were excluded due to the outcome of interest is not reported, and 2 of them were excluded due to inaccessibility of the full text. As a result, 19 studies were met the inclusion criteria to undergo the final systematic review and meta-analysis (Figure 1).

Studies were conducted from different regions of Ethiopia (Amhara, Oromia, SNNPR (South Nation Nationalities people and representatives), Addis Ababa (AA), and Tigray). Totally, ten thousand one hundred twenty one (10,121) women living with HIV/AIDS were included in this systematic review and meta-analysis. In this review, four studies were from Addis Ababa [14, 23–25], three studies from South Nation Nationalities and people representatives [16, 26, 27], two studies from Tigray region [15, 28], eight studies from Amhara region [13, 17, 29–34], and two were from Oromia region [35, 36]. Regarding the sample size, in range from 308-1418 HIV positive women were recruited for the study with a maximum and minimum contraceptive use report 30.14 percent to 93.63 percent, respectively (Table 2).

### 3.2. Contraceptive Use among HIV Positive Women in Ethiopia

**Figure 2:**
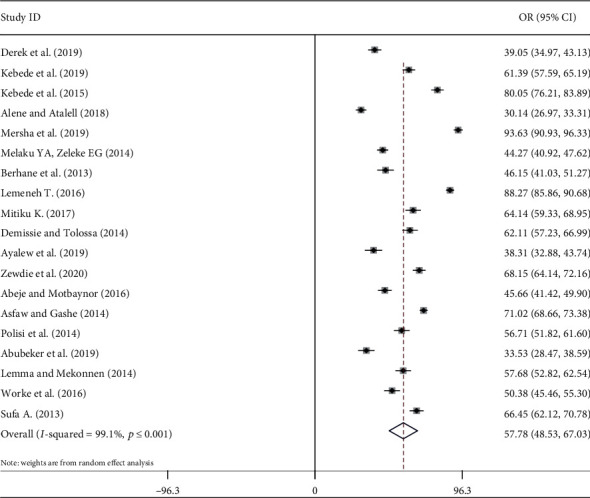
Forest plot of the pooled prevalence of contraceptive use among HIV positive women in Ethiopia.

In this study, the national estimated prevalence of contraceptive use in Ethiopia was 57.78% (95% CI 48.53-67.03) (Figure 2).

### 3.3. Preference of Current Contraceptive Use among HIV Positive Women

In this systematic review and meta-analysis, the commonest preferred contraceptive use among women living with HIV/AIDS was injectable 36.00% (95% CI: 26.64-45.35), male condom 32.74% (95% CI: 21.08-44.40), and implants 13.10% (95% CI: 8.82-17.38) (Table 3).

### 3.4. Heterogeneity and Publication Bias

**Figure 3:**
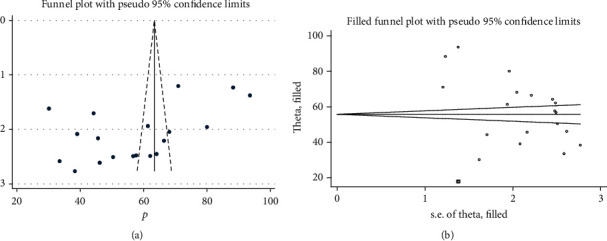
(a) Funnel plot to test publication bias of 19 studies. (b) Result of trim and fill analysis for adjusting publication bias of the 20 studies.

In this meta-analysis, we execute heterogeneity within the included studies, indicating the presence of considerable heterogeneity (*I*^2^ = 99.1%, *p* < 0.001). We also assessed the presence of publication bias by using Egger's test, which suggests the presence of publication bias (*p* = 0.012). As a result, trim and fill analysis was conducted to overcome the publication bias. After one study was filled, twenty studies were enrolled and computed through the trim and fill analysis with a pooled prevalence of 59.26% (95% CI: 58.44-60.08) using a random effect model (Figures 3(a) and 3(b)).

### 3.5. Sensitivity Analysis

In this meta-analysis, to investigate the potential source of heterogeneity observed in pooled prevalence of contraceptive use, a leave-one-out sensitivity analysis was executed and suggesting that our findings was not dependent on a single study. The pooled prevalence of contraceptive use among HIV positive women was varied between 55.78% (47.13-64.43%) and 59.33% (50.59-68.07%) after deletion of a single study (Table 4).

### 3.6. Subgroup Analysis

We conducted a subgroup analysis based on the region where the study was conducted and year and sample size. With regard to sample size, the highest prevalence was observed among those studies with sample size of less than 500, 59.07% (95% CI: 46.88-71.25). Regarding the region of study conducted, the highest prevalence was observed in studies done in Oromia region with 61.65% (95% CI: 52.10-71.19), and the lowest was in Tigray with 44.83% (95% CI: 42.03-47.63) (Table 5).

### 3.7. Determinants of Contraceptive Use

In this systematic review and meta-analysis, a total of nineteen primary studies [19] with data that can be analyzed, we have examined the associations of determinants (discussion with husband/partner, HIV status disclosure, ever counseled on modern contraceptives, having more than one child and marital status) with contraceptive use among women living with HIV/AIDS.

**Figure 4:**
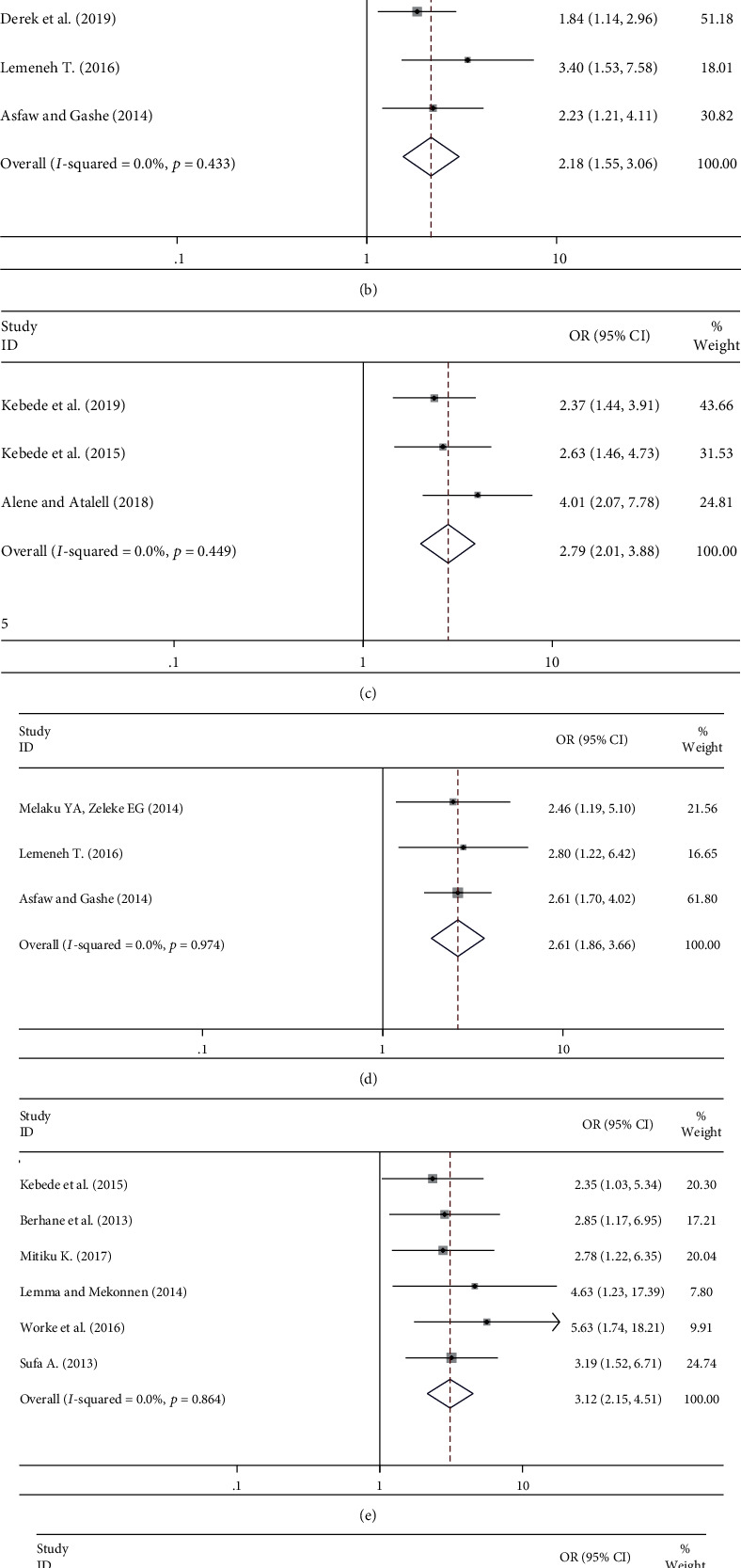
Forest plot showing the pooled odds ratio of the associations between contraceptive use and its determinants: (a) discussion with partner, (b) disclosing HIV status, (c) counseling on contraceptive, (d) having more than one child, (e) secondary and above education, and (f) being widowed/separated.

In this meta-analysis, mothers who have discussed with their husband/parent were 4.7 times more likely to use contraceptives as compared with mothers who have not discussed with their husband/parent with odds ratio (AOR: 4.70, 95% CI: 2.18-10.12) (Figure 4(a)).

HIV status disclosure was also another determinant factor associated with contraceptive use. Mothers who disclose their HIV status to their spouse/parent were 2.18 times more likely to utilize contraceptives as compared with mothers who did not disclose their HIV status to their spouse/parent (AOR: 2.18, 95% CI: 1.55-3.06) (Figure 4(b)).

Those mothers who ever counseled about modern contraceptives were 2.79 times more likely to use contraceptives as compared with their counter parts (AOR: 2.79, 95% CI: 2.01-3.88) (Figure 4(c)). Similarly, mothers who have more than one live child were 2.61 times more likely to utilize contraceptives as compared with mothers who have one child (AOR: 2.61, 95% CI: 1.86-3.66) (Figure 4(d)).Those women living with HIV/AIDS who completed secondary and above education were 3.12 times more likely to use contraceptives as compared with their counter parts (AOR: 3.12, 95% CI: 2.15-4.51) (Figure 4(e)). But in this meta-analysis, the odds of contraceptive use among currently unmarried women were 77% less likely as compared with currently married women (AOR: 0.23, 95% CI: 0.16-0.34) (Figure 4(f)).

## 4. Discussion

Modern contraceptive utilization among HIV positive mothers added the benefit of preventing unintended pregnancies, reducing the need for unsafe abortions, and HIV positive births and can have a major impact on improving the overall maternal and infant health. Therefore, this systematic review and meta-analysis is aimed at estimating the pooled prevalence of contraceptive use among HIV positive mothers and its associated factors in Ethiopia. In this meta-analysis, the pooled prevalence of contraceptive use among HIV positive mothers in Ethiopia was 57.78% (95% CI: 48.53-67.03). Even though there was no analogous meta-analysis study conducted on this specific research question within the area, the pooled prevalence of contraceptive use among HIV positive mothers is comparable with a survey done in Nairobi, Kenya (55.5%) [37], Northern Tanzania (54%) [38], Uganda (58%) [5], and Swaziland (62.5%) [39] (Warren et al. 2013). This is higher than a survey conducted in Kenya (32.3%) [40] and Northern Uganda (25%) [41]. But, it is lower than a study conducted in South Africa (78%) [42], Togo (73.1%) [43], Southeast Nigeria (73.1%) [44], and Zambia (69%) [45]. This difference might be due to regional variation and variation in guidelines of reproductive health and health policy of the country.

In this meta-analysis concerning method of contraceptive preference, the most widely preferred contraceptive method was injectable for 36.00% (95% CI: 26.64-45.35) and male condom for 32.74% (95% CI: 21.08-44.40). This is supported with a study conducted in Northern Tanzania [38]. This might be due to the fact that most HIV positive women preferred short acting contraceptive methods to conceive within a short period of time due to high pregnancy desire.

This meta-analysis showed that women who attended secondary and above education were more likely to utilize contraceptive as compared with their counter parts. This is supported with a study done in Northern Uganda [41] and Lusaka, Zambia [45]. This might be due to the fact that education improves HIV/AIDS knowledge and communication with partner as well as increasing female decision-making power on reproductive health particularly family planning, thus enabling them to easily change risky sexual behavior. Mothers who have discussed with their husband/parent were more likely to use contraceptives as compared with mothers who have not discussed with their husband/parent. This is consistent with a study done in Northern Uganda [41], Northern Tanzania [38], and India [46]. This might be due to the fact that male has higher decision-making power, and currently in Ethiopia, male involvement in PMTCT and related reproductive health services became increased which ultimately increase contraceptive usage to minimize unintended pregnancy. Similarly, mothers who disclosed their HIV status to their spouse/parent were more likely to utilize contraceptives as compared with mothers who did not disclosed their HIV status to their spouse/parent. This is in line with a study done in Uganda [5], Canada [47], and Zambia [45]. This might be due to nowadays stigmatization, and misconception-related HIV/AIDS was reduced due to community awareness and raised spousal support which leads HIV positive women to disclose their status to their spouse/parent.

Those mothers who ever counseled about modern contraceptives were more likely to use contraceptives as compared with their counter parts. This is consistent with a study conducted in Northern Tanzania [38]. This might be explained by HIV positive women who receive more information on reproductive health since they are frequently in contact with health care providers during their follow-up visit. Additionally, nowadays, the government gives great emphasis on couple counseling about contraceptive methods to minimize the occurrence of unintended pregnancy due to disapproval of the husband.

Contraceptive use was higher among mothers who have more than one living child as compared with mothers who have one child. This finding is supported with a study conducted in Nairobi, Kenya [37]. This might be explained by women with more than one living child that might use contraceptive methods to space or limit child births. In fact, those women who have already reached the number of children they want used contraceptive methods. But in this meta-analysis, the odds of contraceptive use among currently unmarried women were less likely as compared with currently married women. This is in line with a study done in Nairobi, Kenya [37], and Swaziland (62.5%) [39]. This might be explained that they did not use contraceptive methods due to the fact that they were free from sexual intercourse.

### 4.1. Limitations of the Study

The study designs for all primary studies included in this systematic review and meta-analysis were cross-sectional; as the result, the confounding variables most of the time might affect the outcome variable. Despite, generalizability is possible since the study is at national level; however, establishing temporal cause and effect relationship is impossible due to the natural design limitation of cross-sectional studies.

## 5. Conclusion

In this study, nearly three-fifth of HIV positive women used contraceptive methods. HIV status disclosure, attending secondary and above education, women discussed with their spouse/parent, having more than one living child, and ever counselled about contraceptive methods were factors that increase the likelihood of contraceptive use among HIV positive women. Therefore, based on the study findings, husband involvement and providing counselling about contraceptive methods for HIV positive women should be encouraged, and there is a need for an intensified effort to improve reproductive health service utilization. Health care providers in charge of HIV care giving must also integrate family planning services in HIV care during follow-up visits.

## Figures and Tables

**Table 1:** Search for MEDLINE/PubMed and Google Scholar databases to assess contraceptive use among HIV positive women in Ethiopia.

Databases	Searching terms	Number of studies
MEDLINE/PubMed	“Contraceptive use” OR “uptake of contraceptive” OR “utilization of family planning,” OR “modern contraceptive use” OR “contraceptive preference,” OR “met need of family planning,” OR “uptake of family planning” AND “among women living with HIV/AIDS,” OR “among sexually active women living with HIV/AIDS”	356
Google scholar	“Contraceptive use” or “modern contraceptive use” and “determinants” or “associated factors” and “HIV positive women” and “Ethiopia”	138
From other databases		83
Total retrieved articles		577
Number of included studies		19

**Table 2:** Study characteristics included in the systematic review and meta-analysis.

Authors	Publication year	Region	Study design	Sample size	%	Contraceptive preference (%)					
						Pill	Injectable	Implant	IUD	Condom	Quality
Derek et al. [12]	2019	AA	C-S	548	39.05	8.10	17.20	—	18.00	42.60	Low risk
Kebede et al. [29]	2019	Amhara	C-S	632	61.39	9.30	51.50	29.40	2.80	7.00	Low risk
Kebede et al. [30]	2015	Amhara	C-S	416	80.05	6.70	54.70	17.70	1.80	52.00	Low risk
Alene and Atalell [10]	2018	Amhara	C-S	803	30.14	4.90	42.80	11.10	6.20	32.90	Low risk
Mersha et al. [31]	2019	Amhara	C-S	314	93.63	7.00	25.80	5.10	28.40	26.70	Low risk
Melaku YA and Zeleke EG [28]	2014	Tigray	C-S	847	44.27	2.70	70.70	8.60	—	47.60	Low risk
Berhane et al. [11]	2013	Tigray	C-S	364	46.15	4.20	22.70	2.40	19.00	—	Low risk
Lemeneh T. [23]	2016	AA	C-S	682	88.27	9.40	32.00	24.70	9.80	21.30	Low risk
Mitiku K. [13]	2017	SNNPR	C-S	382	64.14	10.20	64.90	9.80	2.40	31.40	Low risk
Demissie and Tolossa [26]	2014	SNNPR	C-S	380	62.11	5.50	26.30	3.80	—	90.70	Low risk
Ayalew et al. [32]	2019	Amhara	C-S	308	38.31	—	—	—	—	—	Low risk
Zewdie et al. [33]	2020	Amhara	C-S	518	68.15	8.00	32.60	35.90	14.20	8.20	Low risk
Abeje and Motbaynor [34]	2016	Amhara	C-S	530	45.66	7.40	74.40	3.70	4.50	10.30	Low risk
Asfaw and Gashe [24]	2014	AA	C-S	1418	71.02	—	30.50	—	—	45.70	Low risk
Polisi et al. [35]	2014	Oromia	C-S	395	56.71	8.90	11.20	9.40	2.70	34.40	Low risk
Abubeker et al. [25]	2019	AA	C-S	334	33.53	13.40	22.30	33.00	6.30	20.50	Low risk
Lemma and Mekonnen [27]	2014	SNNPR	C-S	397	57.68	1.80	8.80	3.50	0.80	35.80	Low risk
Worke et al. [14]	2016	Amhara	C-S	397	50.38	12.40	48.50	16.50	1.50	8.20	Low risk
Sufa A. [36]	2013	Oromia	C-S	456	66.45	4.30	11.70	1.70	18.00	41.60	Low risk

C-S: cross-sectional; SNNPR: Southern nation nationalities and peoples representatives; AA: Addis Ababa.

**Table 3:** Contraceptive method preference of HIV positive women in Ethiopia.

Type of post abortion family planning methods	Pooled prevalence 95%	*I*-squared
Oral contraceptive pills (OCP)	6.91 (5.38-8.44)	79.9, *p* < 0.001
Injectable	36.00 (26.64-45.35)	98.6, *p* < 0.001
Implants	13.10 (8.82-17.38)	97.0, *p* < 0.001
Intrauterine devices (IUD)	7.76 (5.13-10.38)	94.7, *p* < 0.001
Male condom	32.74 (21.08-44.40)	99.2, *p* < 0.001

**Table 4:** Sensitivity analysis of the prevalence of contraceptive use among HIV positive women in Ethiopia.

Study omitted	Prevalence	95% CI
Derek et al. (2019)	58.82	49.42-68.23
Kebede et al. (2019)	57.57	47.79-67.36
Kebede et al. (2015)	56.54	46.94-66.14
Alene and Atalell (2018)	59.33	50.59-68.07
Mersha et al. (2019)	55.78	47.13-64.43
Melaku YA and Zeleke EG (2014)	58.53	48.99-68.07
Berhane et al. (2013)	58.42	48.87-67.97
Lemeneh T. (2016)	56.07	47.12-65.03
Mitiku K. (2017)	57.43	47.74-67.11
Demissie and Tolossa (2014)	57.54	47.86-67.21
Ayalew et al. (2019)	58.85	49.41-68.29
Zewdie et al. (2020)	57.20	47.46-66.94
Abeje and Motbaynor (2016)	58.45	48.89-68.02
Asfaw and Gashe (2014)	57.03	46.94-67.12
Polisi et al. (2014)	57.84	48.18-67.49
Abubeker et al. (2019)	59.12	49.78-68.45
Lemma and Mekonnen (2014)	57.78	48.12-67.45
Worke et al. (2016)	58.19	48.58-67.79
Sufa A. (2013)	57.29	47.58-67.01

**Table 5:** Subgroup analysis of contraceptive use among HIV positive women in Ethiopia.

Variables	Characteristics	Included studies	Number of study participants	Prevalence with (95% CI)	*I* ^2^, *p* value
Sample size	>500	8	5978	56.02 (41.05-70.98)	99.4, <0.001
	<500	11	4143	59.07 (46.88-71.25)	98.8, <0.001
Region	AA	4	2982	58.05 (34.99-81.11)	99.5, <0.001
	Amhara	8	3918	58.49 (41.16-75.82)	99.4, <0.001
	Oromia	2	851	61.65 (52.10-71.19)	88.3, =0.003
	SNNPR	3	1159	61.32 (57.58-65.06)	44.1, =0.167
	Tigray	2	1211	44.83 (42.03-47.63)	—
Overall		19	10,121	57.78 (48.53-67.03)	99.1, <0.001

## Data Availability

All related data has been presented within the manuscript. The dataset supporting the conclusions of this article is available from the authors on request.

## Abbreviations

AIDS: Acquired immunodeficiency syndrome
AOR: Adjusted odds ratio
ART: Antiretroviral treatment
CI: Confidence interval
EDHS: Ethiopian Demographic and Health Survey
HIV: Human immunodeficiency virus
IUD: Intrauterine device
WHO: World Health Organizations.

## Supplementary Materials

Supplementary Materials Additional file: Table S1: NOS Checklist tool for included studies.

## References

[1] UNAIDS (2019). *Global HIV & AIDS statistics — 2019 fact sheet*.

[2] Adeniyi O. V., Ajayi A. I., Moyaki M. G., Ter Goon D., Avramovic G., Lambert J. (2018). High rate of unplanned pregnancy in the context of integrated family planning and HIV care services in South Africa. *BMC health services research*.

[3] Munsakul W., Lolekha R., Kowadisaiburana B. (2015). Dual contraceptive method use and pregnancy intention among people living with HIV receiving HIV care at six hospitals in Thailand. *Reproductive health*.

[4] Central Statistical Agency (CSA) [Ethiopia] and ICF (2018). *Ethiopia Demographic and Health Survey 2016: HIV Report*.

[5] Wanyenze R. K., Tumwesigye N. M., Kindyomunda R. (2011). Uptake of family planning methods and unplanned pregnancies among HIV-infected individuals: a cross-sectional survey among clients at HIV clinics in Uganda. *Journal of the International AIDS Society*.

[6] WHO (2010). *World Health Organization; Antiretroviral Drugs for Treating Pregnant Women and Preventing HIV Infection in Infants: Recommendations for a Public Health Approach*.

[7] Reynolds H. W., Janowitz B., Homan R., Johnson L. (2006). The value of contraception to prevent perinatal HIV transmission. *Sexually transmitted diseases*.

[8] WHO (2010). *World Health Organizations; PMTCT strategic vision 2010–2015 : preventing mother-to-child transmission of HIV to reach the UNGASS and Millennium Development Goals*.

[9] Central Statistical Agency (CSA) (Ethiopia) and ICF (2016). *Ethiopia Demographic and Health Survey 2016*.

[10] Alene K. A., Atalell K. A. (2018). Contraceptive use and method preference among HIV-positive women in Amhara region, Ethiopia. *BMC women's health*.

[11] Berhane Y., Berhe H., Abera G. B., Berhe H. (2013). Utilization of modern contraceptives among HIV positive reproductive age women in Tigray, Ethiopia: a cross sectional study. *Isrn Aids*.

[12] Derek A., Seme A., Anye C. S., Nkfusai C. N., Cumber S. N. (2019). Modern family planning use among people living with HIV/AIDS: a facility based study in Ethiopia. *The Pan African Medical Journal*.

[13] Mitiku K., Mulugeta S., Lemessa B. (2017). Modern contraceptive utilization and associated factors among HIV positive women on antiretroviral therapy in Mizan-Tepi teaching and referral hospital South-West Ethiopia. *Journal of Reproductive Health and Contraception*.

[14] Worke M. D., Bezabih L. M., Woldetasdik M. A. (2016). Utilization of contraception among sexually active HIV positive women attending art clinic in University of Gondar Hospital: a hospital based cross-sectional study. *BMC women's health*.

[15] Calvert C., Ronsmans C. (2013). The contribution of HIV to pregnancy-related mortality: a systematic review and meta-analysis. *AIDS*.

[16] Gelagay A. A., Koye D. N., Yeshita H. Y. (2018). Factors affecting long acting and permanent contraceptive methods utilization among HIV positive married women attending care at ART clinics in Northwest Ethiopia. *Archives of Public Health*.

[17] Subedi R., Jahan I., Baatsen P. (2018). Factors influencing modern contraceptive use among adolescents in Nepal. *Journal of Nepal Health Research Council*.

[18] Downes M. J., Brennan M. L., Williams H. C., Dean R. S. (2016). Development of a critical appraisal tool to assess the quality of cross-sectional studies (AXIS). *BMJ Open*.

[19] Song F., Gilbody S. (1998). Bias in meta-analysis detected by a simple, graphical test. Increase in studies of publication bias coincided with increasing use of meta-analysis. *BMJ*.

[20] Sterne J. A., Egger M. (2001). Funnel plots for detecting bias in meta-analysis: guidelines on choice of axis. *Journal of clinical epidemiology*.

[21] Borenstein M., Hedges L. V., Higgins J. P., Rothstein H. R. (2010). A basic introduction to fixed-effect and random-effects models for meta-analysis. *Research synthesis methods*.

[22] Rücker G., Schwarzer G., Carpenter J. R., Schumacher M. (2008). Undue reliance on I 2 in assessing heterogeneity may mislead. *BMC medical research methodology*.

[23] Seme T. L. A. (2016). *Contraceptive utilization and associated factors among hHIV positive women enrolled in HIV care in health centers of Addis Ababa*.

[24] Asfaw H. M., Gashe F. E. (2014). Contraceptive use and method preference among HIV positive women in Addis Ababa, Ethiopia: a cross sectional survey. *BMC Public Health*.

[25] Abubeker F. A., Fanta M. B., Dalton V. K. (2019). Unmet need for contraception among HIV-positive women attending HIV care and treatment service at Saint Paul’s Hospital Millennium Medical College, Addis Ababa, Ethiopia. *International journal of reproductive medicine*.

[26] Demissie B., Tolossa D. (2014). Contraceptive use among HIV-infected women attending treatment and Care at Yirgalem Hospital, Southern Ethiopia. *Eastern Africa Social Science Research Review*.

[27] Lemma L. (2014). *Assessment of utilization of modern family planning methods among women living with HIV/AIDS who are on chronic care follow up in Yirgalem public health facilities*.

[28] Melaku Y. A., Zeleke E. G. (2014). Contraceptive utilization and associated factors among HIV positive women on chronic follow up care in Tigray Region, Northern Ethiopia: a cross sectional study. *PloS one*.

[29] Kebede Y. B., Geremew T. T., Mehretie Y., Abejie A. N., Bewket L., Dellie E. (2019). Associated factors of modern contraceptive use among women infected with human immunodeficiency virus in Enemay District, Northwest Ethiopia: a facility-based cross-sectional study. *BMC Public Health*.

[30] Kebede H. G., Ababa A., Ababa A., Ababa A. (2015). Assessment of contraceptive use and associated factors among HIV positive women in Bahir-Dar Town Northwest Ethiopia. *Open Access Library Journal*.

[31] Mersha A. G., Erku D. A., Belachew S. A., Ayele A. A., Gebresillassie B. M., Abegaz T. M. (2019). Contraceptive use among HIV-positive and negative women: implication to end unintended pregnancy. *Contraception and reproductive medicine*.

[32] Tewabe T., Abdanur A., Jenbere D., Ayehu M., Talema G. (2019). Contraceptive use and associated factors among sexually active HIV positive women attending ART clinic in FHRH in Bahir Dar, north west, Ethiopia, 2018. *Facility based cross-sectional study*.

[33] Zewdie Z., Yitayal M., Kebede Y., Gebeyehu A. (2020). Status of family planning integration to HIV care in Amhara regional state, Ethiopia. *BMC pregnancy and childbirth*.

[34] Abeje G., Motbaynor A. (2016). Demand for family planning among HIV positive women on ART: the case of South Gondar and North Wollo Zones Amhara region. *BMC research notes*.

[35] Polisi A., Gebrehanna E., Tesfaye G., Asefa F. (2014). Modern contraceptive utilization among female ART attendees in health facilities of Gimbie town, West Ethiopia. *Reproductive health*.

[36] Sufa A., Abera M., Admasu B. (2013). Utilization of family planning methods and associated factors among women living with HIV attending ART clinics in Nekemte public health facilities, East Wollega Zone, Ethiopia. *Science, Technology and Arts Research Journal*.

[37] Wekesa E., Coast E. (2015). Contraceptive need and use among individuals with HIV/AIDS living in the slums of Nairobi, Kenya. *International Journal of Gynecology & Obstetrics*.

[38] Damian D. J., George J. M., Martin E., Temba B., Msuya S. E. (2018). Prevalence and factors influencing modern contraceptive use among HIV-positive women in Kilimanjaro region, northern Tanzania. *Contraception and reproductive medicine*.

[39] Warren C. E., Abuya T., Askew I. (2013). Family planning practices and pregnancy intentions among HIV-positive and HIV-negative postpartum women in Swaziland: a cross sectional survey. *BMC pregnancy and childbirth*.

[40] Magadi M. A., Magadi W. A. (2017). HIV/AIDS and contraceptive use: factors associated with contraceptive use among sexually active HIV-positive women in Kenya. *Contraception*.

[41] Nattabi B., Li J., Thompson S. C., Orach C. G., Earnest J. (2011). Family planning among people living with HIV in post-conflict Northern Uganda: A mixed methods study. *Conflict and Health*.

[42] Kaida A., Laher F., Strathdee S. A. (2010). Contraceptive use and method preference among women in Soweto, South Africa: the influence of expanding access to HIV care and treatment services. *PloS one*.

[43] Yaya I., Patassi A. A., Landoh D. E. (2018). Modern contraceptive use among HIV-infected women attending HIV care centres in Togo: a cross-sectional study. *BMJ Open*.

[44] Ezugwu E. C., Nkwo P. O., Agu P. U., Ugwu E. O., Asogwa A. O. (2014). Contraceptive use among HIV-positive women in Enugu, Southeast Nigeria. *International Journal of Gynecology & Obstetrics*.

[45] Hancock N. L., Chibwesha C. J., Bosomprah S. (2016). Contraceptive use among HIV-infected women and men receiving antiretroviral therapy in Lusaka, Zambia: a cross-sectional survey. *BMC Public Health*.

[46] Dugg P., Chhabra P., Sharma A. K. (2020). Contraceptive use and unmet need for family planning among HIV-positive women: a hospital-based study. *Indian Journal of Public Health*.

[47] Loutfy M. R., Hart T. A., Mohammed S. S. (2009). Fertility desires and intentions of HIV-positive women of reproductive age in Ontario, Canada: a cross-sectional study. *PloS one*.

