# Supplementary material for: Contraceptive Use and Method Preferences among HIV Positive Women in Ethiopia: A Systematic Review and Meta-analysis
Source: Biomed Res Int. 2020 Sep 18;2020:6465242. doi: 10.1155/2020/6465242 (PMC7520013; doi:10.1155/2020/6465242)
Supplement: Supplementary Materials — Additional file: Table S1: NOS Checklist tool for included studies. [file 6465242.f1.pdf]

Table S1. Quality assessment of articles (NOS for cross sectional study)

Studies	Selection				Comparability	Outcome		Total score
	Representativeness (1)	Sample size (1)	Non-respondents (1)	Ascertainment of the exposure (risk factor) (2)	The subjects in different outcome groups are comparable, based on the study design or analysis. Confounding factors are controlled (2)	Assessment of the outcome (2)	Statistical test (1)	
Derek et al.(12)	*	*	*	**	*	**	*	9
Kebede et al.(23)	*	*	*	**	*	*	*	8
Kebede et al.(24)	*	*	*	**	*	**	*	9
Alene and Atalell(10)	*	*	*	*	*	**	*	8
Mersha et al.(25)	*	*	*	**	*	**	*	9
Melaku YA, Zeleke EG(26)	*	*	*	**	*	**	*	9
Berhane et al.(11)	*	*	*	**	*	**	*	9
Lemeneh T.(27)	*	*	*	**	*	**	*	9
Mitiku K.(13)	*	*	*	**	*	**	*	9
Demissie & Tolossa(28)	*	*	*	**	*	**	*	9
Ayalew et al.(29)	*	*	*	*	*	*	*	7
Zewdie et al.(30)	*	*	*	**	*	**	*	8
Abeje & Motbaynor(31)	*	*	*	**	*	**	*	9
Asfaw & Gashe(32)	*	*	*	**	*	*	*	8
Polisi et al.(33)	*	*	*	*	*	**	*	8

Abubeker et al.(34)	*	*	*	**	*	*	*	8
Lemma &Mekonnen(35)	*	*	*	**	*	**	*	9
Worke et al.(14)	*	*	*	*	*	**	*	8
Sufa A.(36)	*	*	*	**	*	**	*	9
